# Awards Recommended by the Committee for the Year 1915

**Published:** 1915-08-07

**Authors:** 


### Awards Recommended by the Committee of Distribution for the Year 1915.

#### THIRTY-TWO GENERAL HOSPITALS.

Charing Cross Hospital, £1,301; French Hospital, £401; Great Northern Central Hospital, £1,572; Guy's Hospital, £2,022; Hampstead and N.W. London Hospital, £881; Italian Hospital, £235; Kensington General Hospital, £74; King's College Hospital, £1,703; London Hospital, £7,062; London Homœopathic Hospital, £713; Phillips' Memorial Homœopathic Hospital, £53; London Temperance Hospital, £708; Metropolitan Hospital, £1,293; Mildmay Hospital, £284; Mildmay Memorial Hospital, £60; Miller Hospital and Royal Kent Dispensary, £545; Poplar Hospital, £677; Prince of Wales's Hospital, £991; Royal Free Hospital, £1,417; St. George's Hospital, £2,551; SS. John and Elizabeth Hospital, £179; St. John's Hospital, Lewisham, £283; St. Mary's Hospital, £2,177; St. Thomas's Hospital, £226; Seamen's Hospital Society, £1,400; the Middlesex Hospital and Convalescent Home, £2,958; University College Hospital, £2,101; Walthamstow, etc., Hospital, £172; Wandsworth, Bolingbroke Hospital, £408; West Ham Hospital, £1,042; West London Hospital, £1,419; Westminster Hospital, £1,618.

#### SIX CHEST HOSPITALS.

City of London Hospital for Diseases of the Chest, Victoria Park, £979; Hospital for Consumption, Brompton, £2,325; Mount Vernon Consumption Hospital, Hampstead, £517; Royal Hospital for Diseases of the Chest, £616; Royal National Sanatorium, £45; Royal National Hospital, Ventnor, £200.

#### TWENTY CHILDREN'S HOSPITALS.

Alexandra Hospital for Hip Disease, £315; Banstead Surgical Home, £24; Barnet Home Hospital, £38; Belgrave Hospital, £262; Cheyne Hospital for Incurable Children, £166; East London Hospital for Children, £939; Evelina Hospital for Sick Children, £242; Home for Incurable Children, £48; Home for Sick Children, Sydenham, £112; Hospital for Sick Children, £1,475; Infants' Hospital, Westminster, £251; Kensington, for Children, and Dispensary, £94; Paddington Green Hospital for Children, £319; Queen's Hospital for Children, £1,343; Royal Waterloo Hospital for Children and Women, £586; St. Mary's Hospital, Plaistow, £320; St. Monica's Hospital, Brondesbury, £85; Victoria Hospital for Children, Chelsea, £700; Victoria Home, Margate, £51; Hospital for Hip Disease, Sevenoaks, £84.

#### SEVEN LYING-IN HOSPITALS.

City of London Lying-in Hospital, £119; Clapham Maternity Hospital and Dispensary, £26; East End Mothers' Home, £84; General Lying-in Hospital, Lambeth, £220; Plaistow Maternity Hospital, £41; Queen Charlotte's Lying-in Hospital, £557; British Home for Mothers and Babies, £50.

#### FIVE HOSPITALS FOR WOMEN.

Chelsea Hospital for Women, £334; Hospital for Women, Soho Square, £465; Grosvenor Hospital for Women and Children, £144; New Hospital for Women, £399; Samaritan Free Hospital, 502.

### TWENTY-FOUR OTHER SPECIAL HOSPITALS.

Middlesex Hospital, Cancer Wing, £221; London Fever Hospital, Islington, £250; St. Mark's Hospital for Fistula, £364; National Hospital for the Diseases of the Heart, £130; Female Lock Hospital, Harrow Road, £418; Hospital for Epilepsy, Paralysis, etc., £247; National Hospital for the Paralysed and Epileptic, £980; West End Hospital for Diseases of the Nervous System, £340; Central London Ophthalmic Hospital, £199; Royal Eye Hospital, £242; Royal London Ophthalmic Hospital, £1,007; Royal Westminster Ophthalmic Hospital, £139; Western Ophthalmic Hospital, £84; Royal National Orthopædic Hospital, £723; Royal Sea Bathing Hospital, Margate, £101; St. John's Hospital for Diseases of the Skin, £50; St. Peter's Hospital for Stone, £147; Central London Throat and Ear Hospital, £42; Throat Hospital, Golden Square, £61; London Throat Hospital, £37; Metropolitan Ear, Nose, and Throat Hospital, £63; Royal Ear Hospital, Dean Street, £112; Royal Dental Hospital of London, £185; National Dental Hospital, £74.

### THIRTY-FOUR CONVALESCENT HOSPITALS.

Metropolitan Convalescent Institution, Walton, £360; Metropolitan Convalescent Institution, Broadstairs, £277; Metropolitan Convalescent Institution, Bexhill, £529; All Saints' Convalescent Hospital, Eastbourne, £400; All Saints' Convalescent Home, St. Leonards, £26; Ascot Priory Convalescent Home, £80; Brentwood Convalescent Home for Children, £16; Charing Cross Hospital Convalescent Home, £58; Chelsea Hospital for Women Convalescent Home, St. Leonards, £55; Deptford Medical Mission Convalescent Home, £17; Mrs. Gladstone's Convalescent Home, Mitcham, £70; Friendly Societies' Convalescent Home, Dover, £120; Hahnemann Convalescent Home, Bournemouth, £20; Hanwell Convalescent Home, £7; Hastings, Fairlight Sanatorium, £20; Herbert Convalescent Home, Bournemouth, £14; Herne Bay, Baldwin-Brown Convalescent Home, £55; Homœopathic Hospital Convalescent Home, £4; Hemel Hempstead Convalescent Home, £19; Mary Yolland Home, £15; Mrs. Kitto's Convalescent Home, Reigate, £46; London Hospital Convalescent Home, Tankerton, £74; Police Seaside Home, Brighton, £40; St. Andrew's Convalescent Home, Clewer, £60; St. Andrew's Convalescent Home, Folkestone, £160; St. John's Home for Convalescent and Crippled Children, £16; St. Joseph's Convalescent Home, Bournemouth, £18; St. Leonards-on-Sea Convalescent Home for Poor Children, £114; Eversfield, St. Leonard's, £8; St. Mary's Convalescent Home, Birchington, £20; St. Mary's Convalescent Home, Broadstairs, £142; St. Mary's Convalescent Home, Shortlands, £17; St. Michael's Convalescent Home, Westgate, £22; Seaside Convalescent Hospital, Seaford, £50.

### TWENTY-FOUR COTTAGE HOSPITALS.

Acton Cottage Hospital, £113; Beckenham Cottage Hospital, £87; Blackheath and Charlton Cottage Hospital, £106; Bromley, Kent, Cottage Hospital, £109; Bushey Heath Cottage Hospital, £39; Canning Town Cottage Hospital, £107; Chislehurst, Sidcup, and Cray Cottage Hospital, £67; Coldash Cottage Hospital, £41; Ealing Cottage Hospital, £171; East Ham Cottage Hospital, £71; Eltham Cottage Hospital, £55; Enfield Cottage Hospital, £100; Epsom and Ewell Cottage Hospital, £56; Hounslow Cottage Hospital, £57; Livingstone, Dartford, Cottage Hospital, £127; Kingston, Victoria Hospital, £67; Reigate and Redhill Hospital, £115; Sidcup Cottage Hospital, £33; Teddington, £100; Tilbury (Passmore Edwards) Cottage Hospital, £67; Willesden Cottage Hospital, £102; Wimbledon Cottage Hospital, £64; Wimbledon Nelson Hospital, £91; Wood Green Cottage Hospital, £84.

### ELEVEN INSTITUTIONS.

Florence Nightingale Hospital for Invalid Gentlewomen, £133; St. Saviour's Hospital and Nursing Home, £74; Invalid Asylum, Stoke Newington, £36; Firs Home, Bournemouth, £20; St. Columba's Hospital, £302; Free Home for Dying, Clapham, £120; St. Luke's House, Pembridge Square, £141; Santa Claus Home, Highgate, £74; Royal Mineral Water Hospital, Bath, £60; Winifred House, Holloway, £55; Highgate Convalescent Home, £44.

### FORTY-SEVEN DISPENSARIES.

Battersea Provident Dispensary, £168; Billingsgate Dispensary, £51; Brixton, etc., Dispensary, £46; Brompton Provident Dispensary, £16; Buxton Street Dispensary, £9; Camberwell Provident Dispensary, £40; Child's Hill Provident Dispensary, £6; City Dispensary, £69; Clapham General and Provident Dispensary, £19; Eastern Dispensary, £38; East Dulwich Provident Dispensary, £49; Farringdon General Dispensary, £39; Finsbury Dispensary, £54; Forest Hill Provident Dispensary, £37; Greenwich Provident Dispensary, £27; Hampstead Provident Dispensary, £40; Islington Dispensary, £43; Islington Medical Mission, £56; Kentish Town Medical Mission, £22; Kilburn, Maida Vale, and St. John's Wood Dispensary, £37; Kilburn Provident Medical Institution, £45; London Dispensary, Spitalfields, £13; London Medical Mission, Endel Street, £80; Margaret Street, for Consumption, £28; Metropolitan Dispensary, £25; Mildmay Medical Mission Dispensary, £11; Notting Hill Provident Dispensary, £21; Paddington Provident Dispensary, £33; Public Dispensary, £38; Queen Adelaide's Dispensary, £19; Royal General Dispensary, £21; Royal Pimlico Provident Dispensary, £20; Royal South London Dispensary, £39; St. George's, Hanover Square, Dispensary, £29; St. John's Wood Provident Dispensary, £45; St. Marylebone General Dispensary, £64; St. Pancras and Northern Dispensary, £41; South Lambeth, Stockwell, and North Brixton Dispensary, £26; Stamford Hill, Stoke Newington Dispensary, £60; Tower Hamlets Dispensary, £39; Wandsworth Common Provident Dispensary, £8; Westbourne Provident Dispensary, £15; Western Dispensary, £54; Western General Dispensary, £80; Westminster General Dispensary, £36; Whitechapel Provident Dispensary, £24; Woolwich Provident Dispensary, £32.

### THIRTY-TWO NURSING ASSOCIATIONS.

Belvedere, Abbey Wood, £6 2s. 6d.; Brixton, £30 12s. 6d.; Central St. Pancras, £30 12s. 6d.; Chelsea and Pimlico, £24 10s.; Hackney, £30 12s. 6d.; Hammersmith, £61 5s.; Hampstead, £18 7s. 6d.; Isleworth, £12 5s.; Kensington, £49; Kilburn, £12 5s.; Kingston, £42 17s. 6d.; Lambeth Road (Catholic), £18 7s. 6d.; Metropolitan (Bloomsbury), £49; St. Olave's (Bermondsey), £30 12s. 6d.; Paddington and Marylebone, £55 2s. 6d.; Plaistow, £91 17s. 6d.; Plaistow (Maternity), £171 10s.; Ponders End, Enfield, etc., £12 5s.; Rotherhithe, £12 5s.; Shoreditch, £61 5s.; Sick Room Helps Society, £24 10s.; Sidcup, £6 2s. 6d.; Silvertown, £18 7s. 6d.; South London (Battersea), £55 2s. 6d.; Southwark, £49; South Wimbledon, £30 12s. 6d.; Tottenham, £24 10s.; Westminster, £24 10s.; Woolwich, £42 17s. 6d.; East London, £147; North London, £61 5s.; Ranyard Nurses, £422 12s. 6d.

